# Nitrogen-Mediated Graphene Oxide Enables Highly Efficient Proton Transfer

**DOI:** 10.1038/s41598-017-05570-z

**Published:** 2017-07-12

**Authors:** Guo-Liang Chai, Stephen A. Shevlin, Zhengxiao Guo

**Affiliations:** Department of Chemistry, University College London, London, WC1H 0AJ United Kingdom

## Abstract

Two-dimensional (2D) graphene and graphene oxide (GO) offer great potential as a new type of cost-efficient proton-exchange membranes (PEM) for electrochemical devices. However, fundamental issues of proton transfer mechanism via 2D membranes are unclear and the transfer barrier for perfect graphene are too high for practical application. Using *ab initio* molecular dynamic simulations, we screened the proton transfer barrier for different un-doped and nitrogen doped GO membranes, and clarified the corresponding transfer mechanisms. More significantly, we further identify that N-mediated GO can be built into a highly efficient PEM with a proton transfer rate of seven orders of magnitude higher than an un-doped case via. a proton relay mechanism between a ketone-like oxygen and a pyridine-like nitrogen across the vacancy site. The N-doped 2D GO is also impermeable to small molecules, and hence a highly efficient PEM for practical applications.

## Introduction

Clean and efficient energy technologies are critically important to meet the increasing energy demand and abate undesirable climate change. Redox flow batteries and fuel cells are two of the most promising electrochemical devices for increasing energy efficiency and clean energy applications^1–8^, such as grid integration of renewables and electrification of transport. However, their large-scale applications are hindered by high cost and a limited temperature window of operation, due to expensive electrode catalysts and/or the nature of the polymer membranes.

Currently, the membranes for fuel cells and flow batteries are made from perflourinated proton-exchange polymers^9^. Considerable effort has been devoted to the understanding, control and design of cost-effective membranes^10–17^. However, progress along this direction is slow due to the complicated structures of such membranes. The current benchmark is Nafion, a sulfonated tetrafluoroethylene based fluoropolymer-copolymer discovered in the late 1960s^18–20^. Its porous structure facilitates the migration of cations, e.g. protons, but does not conduct anions. However, Nafion also suffers from poor stability, insufficient resistance to gas crossover and high cost^13^. Generally speaking, polymeric proton-exchange membranes can be classified into three types. The first is perfluorinated membranes and their derivatives modified by metal oxides, such as SiO_2_, TiO_2_ and ZrO_2_, to increase water uptake and proton conductivity at relatively high temperatures^11^. The second is non-fluorinated hydrocarbon (aliphatic or aromatic) polymer membranes, which is less expensive. The third is based on acid-base complexes consisting of an acid component and an alkaline polymer base to promote proton conduction, e.g. phosphoric acid-doped polybenzimidazole (PBI/H_3_PO_4_). In all those cases, proton conductivity strongly depends on the level of water uptake in the membrane. Those also suffer from “cross-over” of gas molecules, such as O_2_ and H_2_. Hence, developing alternative membranes with high performance and low cost is therefore critically important^13^.

Two dimensional (2D) materials such as graphene is versatile and has a lot of applications. A basic issue is that can graphene based material be applied for proton transfer membrane? Recent experiments indicate that two dimensional (2D) graphenes are possible to be a new type of proton-exchange membranes^21, 22^. These studies open up an avenue for the development of efficient 2D proton transfer membranes. However, the proton transfer barrier for an un-doped graphene is too high for effective proton conduction, and the proton transfer mechanism via 2D material is still unclear^21, 22^. Thus, the key challenges are to clarify the mechanisms and identify 2D structure for highly efficient proton conduction membrane for practical application. The current work focuses on the design of a highly efficient single-layer graphene oxide (GO) membrane, as thin GO membranes have been demonstrated to be highly selective for the separation of gases and ions^23, 24^. *Ab initio* molecular dynamic simulations were employed here, which enables the consideration of near-practical conditions, including temperature and an electrolyte environment. The results indicate that the proton transport rate can be increased by seven orders of magnitude if mediated by appropriate nitrogen-doping in 2D GO membranes. This mechanistic study points out a clear direction for experimental development of efficient GO membranes in practice.

## Results

For polymeric membranes, protons may transfer via both Grotthuss (proton hopping) and hydronium ion diffusion mechanisms^11, 25^. The prevalence of one or the other depends on the hydration level of the membrane. Large free spatial sites exist in polymeric chains to facilitate the diffusion of hydronium cations (H_3_O^+^) in an aqueous electrolyte, but this also increases the propensity of gas crossover. In any case, hydronium diffusion via 2D graphene or GO membranes are ruled out experimentally, due to rather small vacancies in such membranes for proton transfer^22^. Therefore, the Grotthuss mechanism should dominate proton transfer via such 2D membranes and is thus the focus of consideration here. *Ab initio* molecular dynamics (AIMD) can explicitly simulate forming and breaking of chemical bonds and hence is the natural choice for this study. AIMD simulations were done using the CPMD code and free energy for proton diffusion calculated by the Blue Moon method.

**Figure 1:**
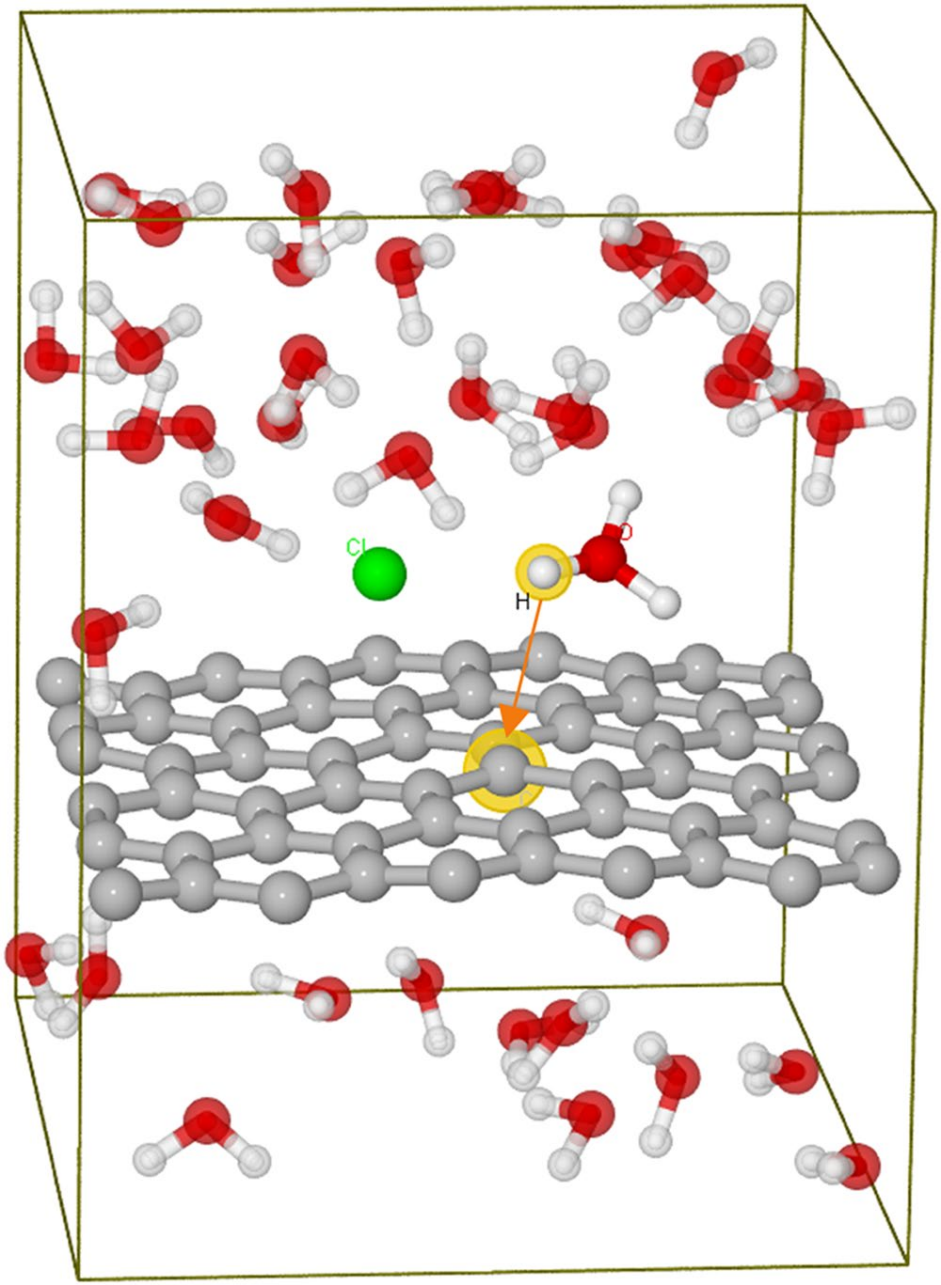
A simulation box of a graphene membrane for calculating PTBs, consisting a single layer graphene in a water solution of hydrochloric acid. The HCl molecule dissociates into a hydronium ion and a chloride ion. The proton is labelled with a yellow halo, which is approaching the C atom with a halo, as pointed by the arrow. The white, red, grey and green spheres are hydrogen, oxygen, carbon and chlorine atoms, respectively.

**Figure 2:**
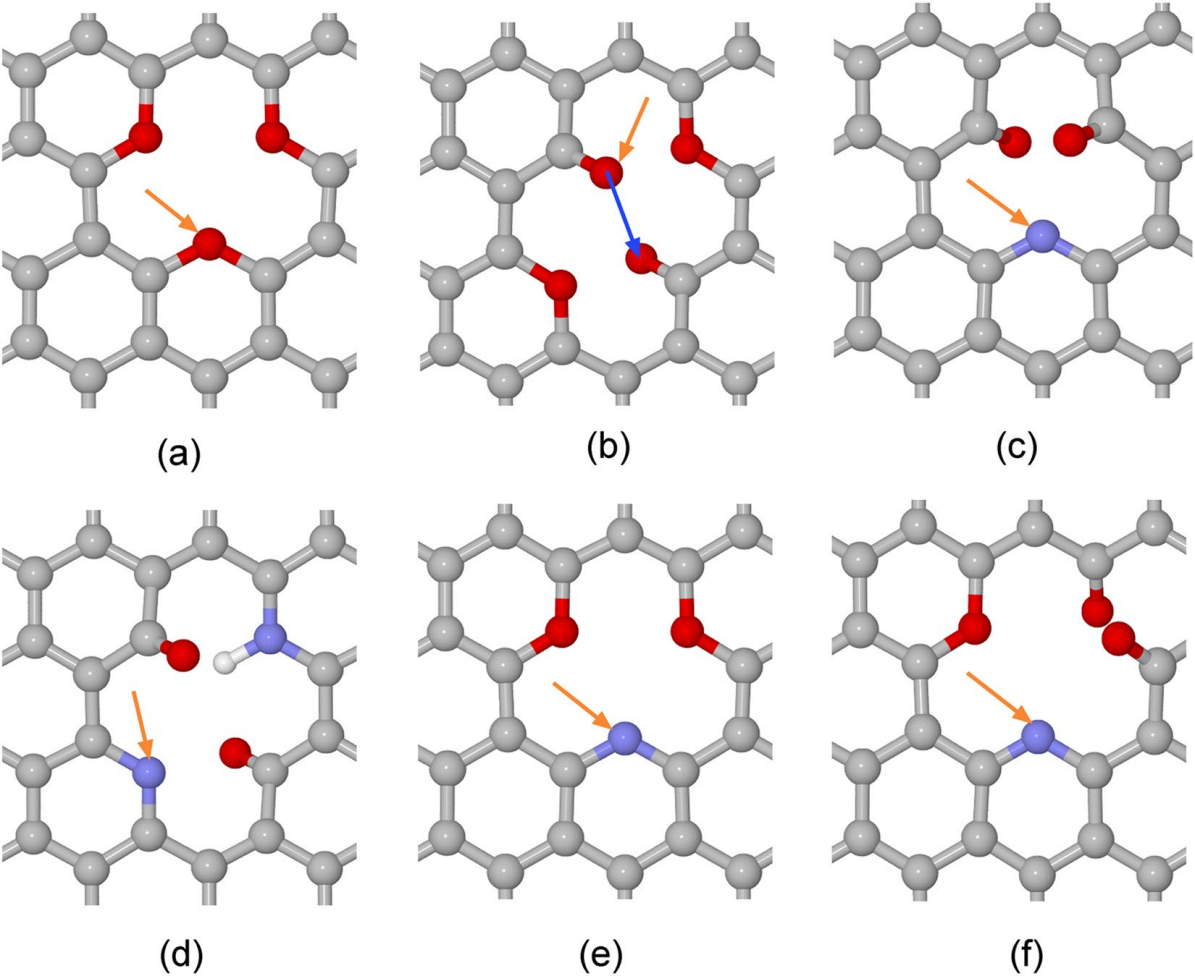
Different local structures considered for proton transport: (**a**) T2-V4-O3, (**b**) T1-V4-O4, (**c**) V2-N1O2, (**d**) T1-V4-N2O2H1, (**e**) T2-V4-N1O2, (**f**) T2-V4-N1O3. NxOyHz: “x”, “y” and “z” are the number of N, O and H atoms at a vacancy site, respectively. “V2” and “V4” are graphene vacancies with two and four carbon atoms removed, respectively. “T1” and “T2” are employed for distinguishing two different types of “V4”. More details are in Supporting Information (SI1). The white, red, grey and blue spheres are hydrogen, oxygen, carbon and nitrogen atoms, respectively. The yellow arrow indicates how a proton transfers from the bulk solution to the surface, and the blue arrow indicates how a proton relays between two sites across the membrane.

Figure 1 shows an example of the simulation box for a graphene membrane in an aqueous electrolyte. The local structures of the studied 2D GO membranes are shown in Fig. 2 and different proton transport pathways are labelled by arrows. The details for the selection of these structures are discussed in Figures S1 to S4 of the Supporting Information (SI1). For comparison, the proton transfer barrier (PTB) for a perfect graphene membrane is also calculated and shown to be at least larger than 3.0 eV, Figure S5 in the Supporting Information (SI2). This barrier is too high for proton transfer at room temperature without added bias. Therefore, we attended to other promising structures, such as un-doped and N-doped 2D GOs, as systematically shown in Fig. 2.

**Figure 3:**
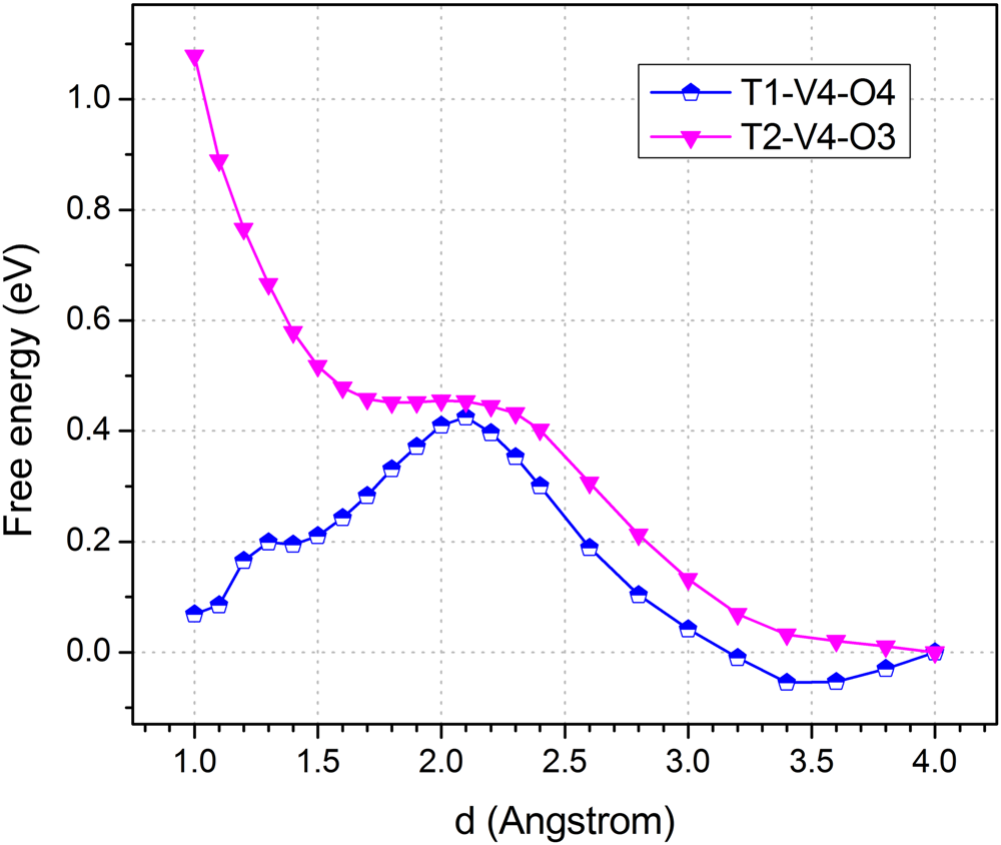
T1-V4-O4 and T2-V4-O3 are proton transport free energy profiles for attachment/detachment barriers of the corresponding structures. Here the “d” along horizontal axis is the distance between the proton and the target site to be attached. Other barriers are summarized in Table 1.

The free energy variations for proton transfer via un-doped GO membranes of different sizes and levels of oxidation are shown in Fig. 3, involving the structures of T2-V4-O3 and T1-V4-O4 in Fig. 2a,b, respectively. The proton transfer free energy profiles for V1-O2, V2-O3 and T2-V4-O4 structures are shown in Figure S7 in Supporting Information (SI3). These are most favorable structures for each type of vacancies according to Figures S1 and S2 in the Supporting Information (SI1). For example, the V1-O2 structure is thermodynamically the most stable, with one pyrylium-like and the another ketone-like oxygen in the (hence oxidized) mono-vacancy; this configuration also agrees with a previous study^26^. Generally speaking, a pyrylium-like oxygen locates in the plane of the membrane surface and is protophobic, while a ketone-like oxygen may provide hydrogen-bonding sites for the water layers^22^.

**Table 1:** PTBs for different 2D structures.

Structure	Barrier (eV)
PG-h	>4.0
PG-C	>3.0
V2-O3	2.13
T1-V4-O4	0.55
T2-V4-O4	1.01
V2-N1O2	0.32
T1-V4-N2O2H1	>0.56
T2-V4-N1O2	0.83
T2-V4-N1O3	0.86

The PG-h and PG-C are for proton transfer through perfect graphene. More details are in Supporting Information (SI2).

For the V1-O2 structure, the barrier for a proton to move from the solution to the ketone-like oxygen is 0.60 eV. Here, the barrier for proton transfer from solution to a surface site is defined as the attachment barrier, and the reverse is the detachment barrier. The barrier for proton relaying between two different surface sites across a vacancy is defined as the relay barrier, which only needs to be considered when the attachment / detachment site involves an “out-of-plane” ketone-like oxygen, as seen in Fig. 2b. For clarity in Fig. 2, the yellow arrow indicates the attachment / detachment site of a proton from/to solution to/from the membrane surface, associated with the attachment and detachment barriers, respectively. Hence, the attachment/detachment barrier can be obtained from the same proton transfer profile for a specific structure as shown in Fig. 3. The process described by the blue arrow corresponds to a proton relay barrier, which only occurs when there is an “out-of-plane” ketone-like oxygen is the attachment/detachment site. Actually, the vacancy of the V1-O2 structure is blocked by the pyrylium-like oxygen, which makes it difficult to relay a proton across the membrane. For the V2-O3 structure, the attachment barrier to a ketone-like oxygen is reduced to 0.43 eV, mainly due to the increase of local electron density around the vacancy to enhance the binding of protons. The two ketone-like oxygen atoms are on two opposite sides of the GO membrane. The proton still needs to transfer across the membrane, as shown by the blue arrow in Fig. 2b, and this attachment barrier for the V2-O3 is as large as 2.13 eV, which is prohibitively high for proton transfer across the membrane.

In order to reduce the proton relay barrier, the vacancy size and the degree of oxidation were continuously increased in the T1-V4-O4, T2-V4-O3 and T2-V4-O4 structures, Fig. 2 and Figure S1. For the T1-V4-O4 structure, there are two pyrylium-like oxygens and alternatingly arranged are two other ketone-like oxygens. This alternating arrangement of pyrylium-like and ketone-like oxygens also makes the structure rather stable. The attachment barrier to the ketone site is 0.42 eV. The corresponding relay barrier for the proton to transfer from one ketone site to the other is 0.55 eV. Therefore, proton relay dominates the proton transport in this case and the overall PTB is 0.55 eV. The T2-V4-O3 structure with three pyrylium-like oxygens is impossible for proton transfer via the Grotthuss mechanism, Fig. 3. For the T2-V4-O4 structure, though the attachment barrier to the ketone site is 0.41 eV, the corresponding relay barrier is as large as 1.01 eV. It is difficult for a proton to transfer through the T2-V4-O4 structure. Hence, the minimum PTB is screened to be 0.55 eV for un-doped oxidized graphnene in the current study for the case of T1-V4-O4.

A previous study indicates that the PTB through 2D GO membranes is about 0.61–0.68 eV by means of hydroxyl terminated graphene vacancies^22^. However, even the optimized barrier of 0.55 eV in the current study is still relatively high for efficient proton transport in practical applications. The proton attachment barrier is slightly reduced from 0.43 to 0.41 eV for V2-O3 and T2-V4-O4 structures, respectively. It is difficult to reduce further this barrier by the increase of the degree of oxidation. Larger sizes of vacancies are not considered further, as it would also increase the possibility of crossover of gas molecules, such as H_2_ and O_2_.

**Figure 4:**
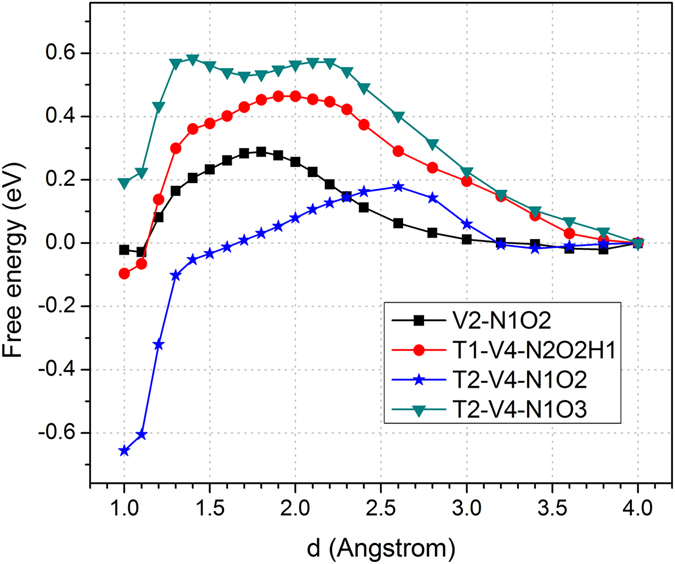
Proton transport free energy profiles for V2-N1O2, T1-V4-N2O2H1, T2-V4-N1O2 and T2-V4-N1O3 structures, respectively. Here the “d” along horizontal axis is the distance between the proton and the target site to be attached.

In order to further reduce the PTB, nitrogen-doping in GO was considered. Nitrogen is selected because it is more electronegative than oxygen and can increase the local electron density and thus propensity for proton attachment. The N-doped GO structures considered here are V2-N1O2, T1-V4-N2O2H1, T2-V4-N1O2 and T2-V4-N1O3, shown in Fig. 2c–f, respectively. The corresponding proton transfer profiles for the N-doped GOs are shown in Fig. 4. The V2-N1O2 structure contains a ketone-like O and pyridine-like N at the vacancy, and the attachment and detachment barriers to the N site are 0.31 eV and 0.32 eV, respectively. Although two C atoms are removed for this structure, it is actually a mono-vacancy as the N atom is a dopant. Due to the symmetry of this structure, the barriers for a proton transfers from the opposite side are symmetric. Therefore, the overall PTB is 0.32 eV for the V2-N1O2 structure. More details about the proton transfer pathway to the N site of the structure are shown in Figure S8 of the Supporting Information (SI4). This small proton shuttling barrier of 0.32 eV makes the V2-N1O2 structure an efficient 2D proton-exchange membrane. Unlike the un-doped 2D GO, such as the T1-V4-O4 structure, for which the proton needs to relay directly from one ketone-like O site to the opposite ketone-like O, the most favorable pathway for the V2-N1O2 structure is for a proton to attach to the ketone-like O first, then move to the N site in plane, and finally transfer to the ketone-like O at the opposite side. As the proton can readily relay between the ketone-like O and pyridine-like N, the PTB is reduced to 0.32 eV, as a result of the N mediation.

For the T1-V4-N2O2H1 structures, there are two N sites around the vacancy, one of which is assumed already hydrogenated and a proton will approach the other N site, because the proton would otherwise be too stable to detach if none of the nitrogen atoms was hydrogenated. The proton attachment and detachment barriers for T1-V4-N2O2H1 are 0.46 and 0.56 eV, respectively. As the barrier of 0.56 eV is already relative large, more details for the T1-V4-N2O2H1 structure are not studied further. In addition, the barriers for H_2_ and O_2_ molecules shuttling across the T1-V4-N2O2H1 structure are calculated to be larger than 2.36 and 8.0 eV, respectively, as shown in the Figure S9 of the Supporting Information (SI5). This means that gas molecule crossover through such a small vacancy is almost impossible.

For the T2-V4-N1O2 structure that contains pyrylium-like O and pyridinic N, the proton attachment barrier to the N site is only 0.18 eV while the detachment barrier is as high as 0.83 eV. Although the proton attachment barrier is reduced, but the proton attached to the N site is too stable to detach. Accordingly, this T2-V4-N1O2 structure is unfavorable for proton conduction. Note here that the calculated barriers are for a proton in solution to attach or transfer to a target site in the membrane surface. If we extrapolate the complete transfer process, the barriers should be symmetrical for an attached proton to detach from the opposite side of the membrane, due to the symmetry of the structure. The figures for the complete permeation process for the T2-V4-N1O2 structure are shown in Figure S10 of the Supporting Information (SI6). Here, we can see that the proton is trapped to the N site due to the large detachment barrier. The vacancy type of the T2-V4-N1O3 structure is similar to that of the T2-V4-N1O2 structure. However, the T2-V4-N1O3 structure possesses two ketone-like oxygens. The proton attachment and detachment barriers to the N site are 0.58 and 0.39 eV, respectively. As this structure is not symmetric for the two opposite sides, the proton attachment and detachment barriers for the other side are 0.86 and 0.43 eV, respectively. This makes the T2-V4-N1O3 structure not efficient for proton transfer.

The overall PTBs for the structures investigated in the present study are summarized in Table 1. According to previous studies, the PTB of a synthesized 2D graphene is ~ 0.78 eV, and the calculated barrier across a hydroxyl terminated vacancy in a graphene membrane is ~ 0.61-0.68 eV^21, 22^. In the current study, the local vacancy structures and the oxidation level of a graphene membrane were tuned for proton transfer, leading to an optimized PTB of about 0.55 eV. More significantly, this PTB can be further reduced to 0.32 eV (V2-N1O2) for nitrogen-doped graphene structures studied here. Generally speaking, different types of defects can co-exist in a graphene^22^. The experimentally observed barriers should be an averaged effect of different sites. Therefore, bottom up synthesis is necessary if the local structures with low PTB are known. In order to understand the reduction of PTBs for different structures, the proton transfer free energy profiles can be roughly divided into three regions: the long distance (>3.0 Å), the short distance (<2.0 Å), and the transition (2.0–3.0 Å) regions. The first region is from the solution to about 3.0 Å to the attachment site, where the barriers for a perfect graphene are almost identical, as shown in Figure S5 of the Supporting Information (SI2). This long distance interaction is mainly due to the hydrophobic properties of the graphene surfaces, which leads to a barrier of about 0.2 eV for a perfect graphene. The local electron density is increased by the introduction of an O or N near the vacancy. For oxidized/N-doped structures in Figs 3 and 4, the barrier to 3.0 Å is reduced to 0.0–0.2 eV due to the reduction of hydrophobicity. For the short distance region within 2.0 Å, hydrogen bonds start to form. For a perfect graphene surface, no hydrogen bond can form between a proton and the membrane surface, which increases the barrier significantly. For the O/N doped graphene, the formation of hydrogen bond reduces the free energy accordingly. And at last the proton bonded to the surface at about 1.0 Å. For the transition region from 3.0 to 2.0 Å, the barrier increases by about another 0.60 eV for a perfect graphene. However, the free energy barrier is only slightly increased in this region for most of the O and N doped vacancies.

The proton transfer rate can be expressed as:

1 $$r=(kT/h).\exp (-{{\rm{\Delta }}}_{r}G/kT).[{H}^{+}]$$

where, *r* is the transfer rate, *k* is Boltzmann constant, *h* is Planck’s constant, *T* is temperature, *Δ*_*r*_ *G* is proton transfer free energy barrier. Hence, the proton transfer rate is increased by seven orders of magnitude (59340345 times) when the free energy barrier is reduced from 0.78 to 0.32 eV at *RT*. Therefore, the high proton conductivity of the V2-N1O2 structure identified in the present study represents a valuable pathway for practical development of highly efficient and cost-effective proton conduction membranes. In fact, the V2-N1O2 structure is a mono-vacancy in graphene as mentioned above. The pyridinic N doped mono-vacancy (V2-N1O2 structure) is relatively easy to create, compared with other types of vacancy clusters or defects. N-doped graphenes can be synthesized, e.g. by way of a bottom-up route from a N-containing precursor or through the post-treatment of graphene or graphite oxide (GO)^27–29^. In fact, pyridine-like or pyridinic N around a mono-vacancy is one of the most observed local structures by means of nitrogen ion bombardment or electron beam irradiation of graphene/graphite^30, 31^. As indicated by previous study, the pyridinic N doped mono-vacancy is one of the most popular structures observed in experiments^30^. In the present study, this structure corresponding to V2-pN. Under an environment with O_2_, the V2-pN structure can be oxidized to a *cis*-V2-N1O2 structure with a low barrier of 0.40 eV, and transfer to the more stable *trans*-V2-N1O2 structure with a barrier about 0.68 eV as shown in Supporting Information (SI6). Further oxidization of *trans*-V2-N1O2 structure is rather difficult with a high barrier of about 4.0 eV. Hence, the V2-N1O2 structure is rather stable.

## Conclusion

In summary, proton transferring via 2D graphene based material is not only a basic issue but also a new type of membrane can be used in electrochemical devices. A study by extensive *ab initio* molecular dynamic simulations shows that the proton transport barrier across an un-doped graphene oxide membrane can be optimized to ~ 0.55 eV for a T1-V4-O4 (di-vacancy with four oxygen atoms) structure. Rather significantly, by further nitrogen doping in the GO membranes, the proton transport barrier can be effectively mediated to 0.32 eV for the V2-N1O2 (mono-vacancy with pyridinic N-doped GO) structure, which makes the proton transfer rate seven orders of magnitude higher than an un-doped case. The carbon, oxygen and nitrogen elements in the GO membranes are all abundant and inexpensive. This pyridinic N-doped GO mono-vacancy should be relatively easy to synthesize via the oxidization of the pyridinic N doped mono-vacancy (V2-pN) as V2-pN is a popular structure in experiments^30^. The study offers great scope for practical development of highly efficient 2D proton-exchange membranes based on graphene oxides.

## Methods

The MD simulation box contains a single layer 2D membrane, HCl molecule and water molecules. The HCl molecule dissociates into hydronium and chloride ions. For one HCl molecule in a simulation box, the concentration of HCl is approximately 1 mol/L. The O-H bond lengths in water molecules are fixed to be the average length of 1.00 Å. Only the proton can move according to Blue Moon method along the transport pathway. For each distance point, we can find that the labelled proton can go through all the possible pathways theoretically (including water-water proton transfer in bulk solution), and automatically choose the best reaction coordinate to approach the membrane surface. Due to the hydrophobic surface of graphene, water molecules are always about 3–4 Å above the surfaces^32, 33^. Thus, the hydronium is placed about 4 Å above the membrane surface as shown in Fig. 1. The proton transport can be described by two mechanisms, that is, Grotthuss mechanism and diffusion mechanism. The Grotthuss mechanism dominates proton transport for 2D materials. Therefore, Car-Parrinello molecular dynamics (MD) simulations were performed to calculate the proton transfer barriers under Grotthuss mechanism^34^. The distance between proton and specific approaching site is constraint by Blue Moon method at 300 K with a time step of 4.0 a.u.^35^. Then constraint force was calculated and hence the free energy barrier. An example of convergence of average force is shown in Figure S13. For each free energy barrier calculation, the total simulation time was about 30 ps. The Becke-Lee-Yang-Parr (BLYP) generalized gradient approximations were employed for the exchange and correlation functionals in spin polarization scheme^36^. Grimme’s semi-empirical method was employed to add van der Waals corrections^37^. The sampling of the Brillouin zone was restricted to the Gamma point. The valence-core interaction was described by Troullier-Martins pseudopotentials (PPs) for C, N, O and H^38^. The plane-wave cutoff energy is set to be 70 Ry.

The total energies were calculated by stationary DFT calculations by employing PWSCF code in Quantum ESPRESSO suite^39^. The GGA-PBE exchange-correlation functional was adopted^40^. Spin polarization was taken into account if it existed. The kinetic energy cutoffs for the wave function and the charge were set to be 35 and 350 Ry, respectively. For the N doped single layer graphene / GO, an orthorhombic supercell with around 60 atoms was used. The barriers under vacuum is calculated by climbing image nudged elastic band (CI-NEB) calculations^41^.

## Electronic supplementary material

Supporting Information
