# Supplementary material for: Nitrogen-Mediated Graphene Oxide Enables Highly Efficient Proton Transfer
Source: Sci Rep. 2017 Jul 12;7:5213. doi: 10.1038/s41598-017-05570-z (PMC5507853; doi:10.1038/s41598-017-05570-z)
Supplement: Supplementary file 1 — Supporting Information [file 41598_2017_5570_MOESM1_ESM.pdf]

Nitrogen-Mediated Graphene Oxide Enables Highly Efficient Proton Transfer

Guo-Liang Chai, Stephen A. Shevlin and Zhengxiao Guo*

Department of Chemistry, University College London, London WC1H 0AJ, United Kingdom

*Email: z.x.guo@ucl.ac.uk

Content

SI1. Structures and Formation Energies of GO and N-Doped GO…… 2

SI2. Proton Transfer Profiles for Perfect Graphene…………………... 8

SI3 Proton Transfer Profiles for V1-O2, V2-O3 and T2-V4-O4……..11

SI4. Pathways for Proton Transport via V2-N1O2 Structure…………11

SI5. H_2/O_2 Molecules Crossover Barriers for T1-V4-N2O2H1 Structure……………………………………………………………….13

SI6. Pathways for Proton Transport via T2-V4-N1O2 Structure……..14

SI7. Discussions for formation of V2-N1O2 structure………………..15

S8. Convergence of calculated force……………………………………..17

SI1. Structures and Formation Energies of GO and N-Doped GO

The structures of different GOs are displayed in **Figure S1**. Five types of GOs are considered as shown in **Figure S1(a)-(e)** for: oxidized perfect graphene, mono-vacancy graphene, di-vacancy graphene and two different graphene tetra-vacancies. For each type of graphene structure, the formation energy of *ith* O in the structure is calculated by:

$$\Delta E_f[(ith)\mathrm{O}] = E[(i)\mathrm{O}] - E[(i\text{-}1)\mathrm{O}] - (i/2)*E(\mathrm{O_2}) \quad \text{(S1)}$$

where i is the number of doped O atoms, $\Delta E_f[(ith)\mathrm{O}]$ is the formation energy of *ith* O on the structure, $E[(i)\mathrm{O}]$ and $E[(i\text{-}1)\mathrm{O}]$ are the total energy of graphene with i and $i\text{-}1$ O atoms. $E(O_2)$ is total energy of O_2, which is corrected by formation energy of water. The corresponding formation energies are shown in **Figure S2.** It can be seen from this figure that the most stable structures under oxygen enrichment are V1-O2 for mono-vacancy, V2-O3 for di-vacancy, T1-V4-O4 for the first type of tetra-vacancy and T2-V4-O4 for the second type of tetra-vacancy. These structures are checked for proton transfer barriers as shown in **Figure 3** of main text. T2-V4-O3 is also checked as the formation energy of 4th O of this structure is not exothermic much. For perfect graphene, the oxidization energy is endothermic, which is not considered for further investigation.

The structures of different N-doped GO are displayed in **Figure S3**. The N doping energy around vacancy is calculated as:

$$\Delta E_d(i\text{N}) = E(i\text{N}) - E_0 + iE(\text{C}) - (i/2)*E(\text{N}_2) \quad \text{(S2)}$$

where i is the number of doped N atoms, $\Delta E_d(i\text{N})$ is the N doping energy, $E(i\text{N})$ is the total energy of N-doped graphene. E_0 is the total energy of un-doped graphene, $E(\text{C})$ is the total energy per atom of perfect graphene. $E(N_2)$ is the total energy of an isolated N_2 molecule. The doping energies for graphitic N and pyridinic N are shown in **Figure S4**, which indicates that the pyridinic N (V2-pN) is much more stable than graphitic N (V2-gN) around vacancy. Hence, only pyridinic N configurations are investigated for oxidization. Oxygen atoms are added to the vacancies until all the dangling C atoms are fully occupied. The oxidization energies are also shown in **Figure S4.** Further oxidization of oxidized C site is rather difficult as shown in Figure S10 in the following. Hence, the most stable N-doped GO structures under oxygen enrichment are V2-N1O2, T1-V4-N2O2, T2-V4-N1O2 and T2-V4-N1O3, respectively. The corresponding proton transfer barriers are calculated and shown in **Figure 4** in main text. Note here that the "V2" structures are actually mono-vacancies because one vacancy site is occupied by N dopant.

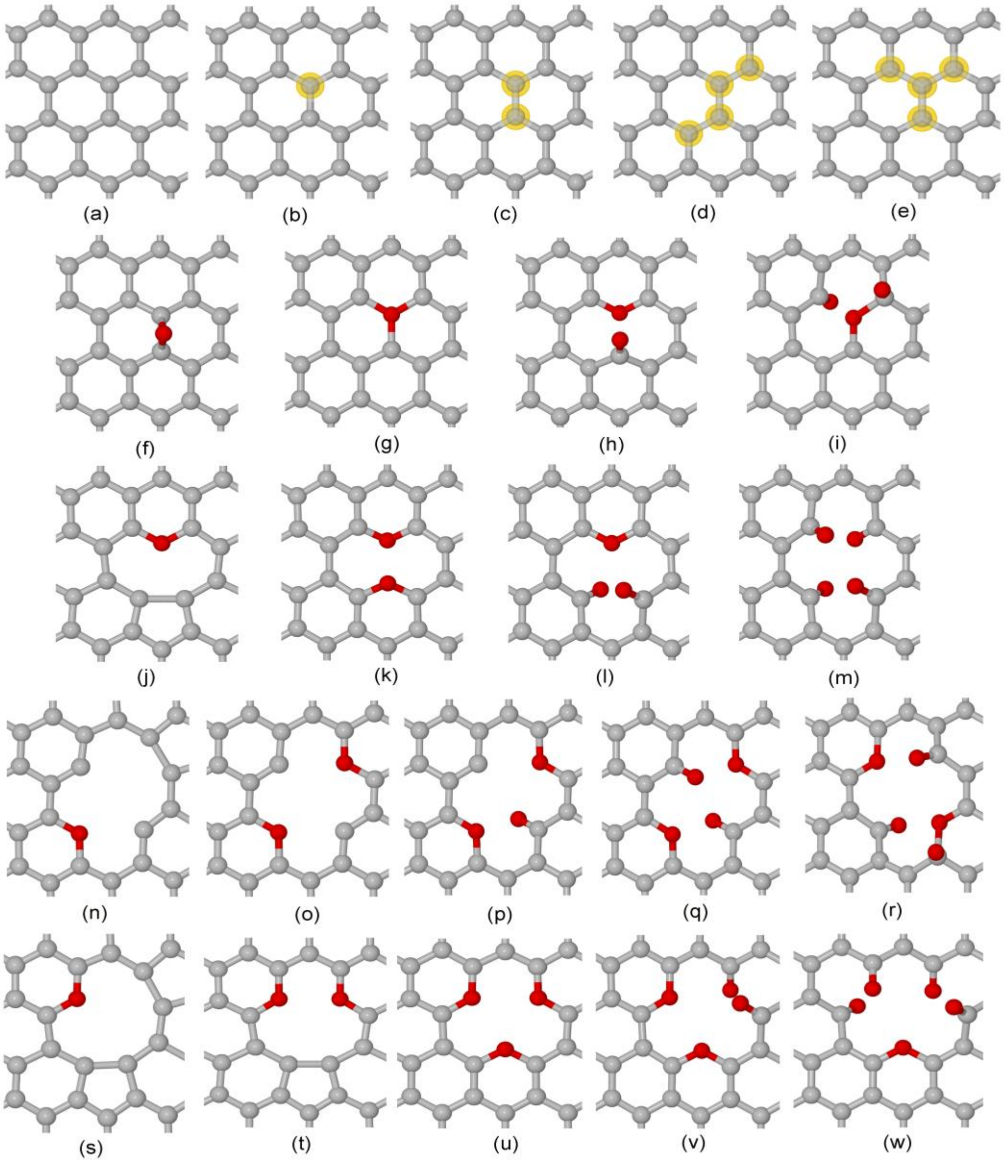

Figure S1. (a) Perfect graphene named "PG", (b) Graphene with one C atom removed vacancy named "V1", (c) Graphene with two C atoms removed vacancy named "V2", (d) The first type of graphene with four C atoms removed vacancy named "T1-V4", (e) The second type of graphene with four C atoms removed vacancy named "T2-V4", (f) PG-O1, (g) V1-O1, (h) V1-O2, (i) V1-O3, (j) V2-O1, (k) V2-O2, (l) V2-O3, (m) V2-O4, (n) T1-V4-O1, (o) T1-V4-O2, (p) T1-V4-O3, (q) T1-V4-O4, (r) T1-V4-O5, (s) T2-V4-O1, (t) T2-V4-O2, (u) T2-V4-O3, (v) T2-V4-O4, (w) T2-V4-O5.

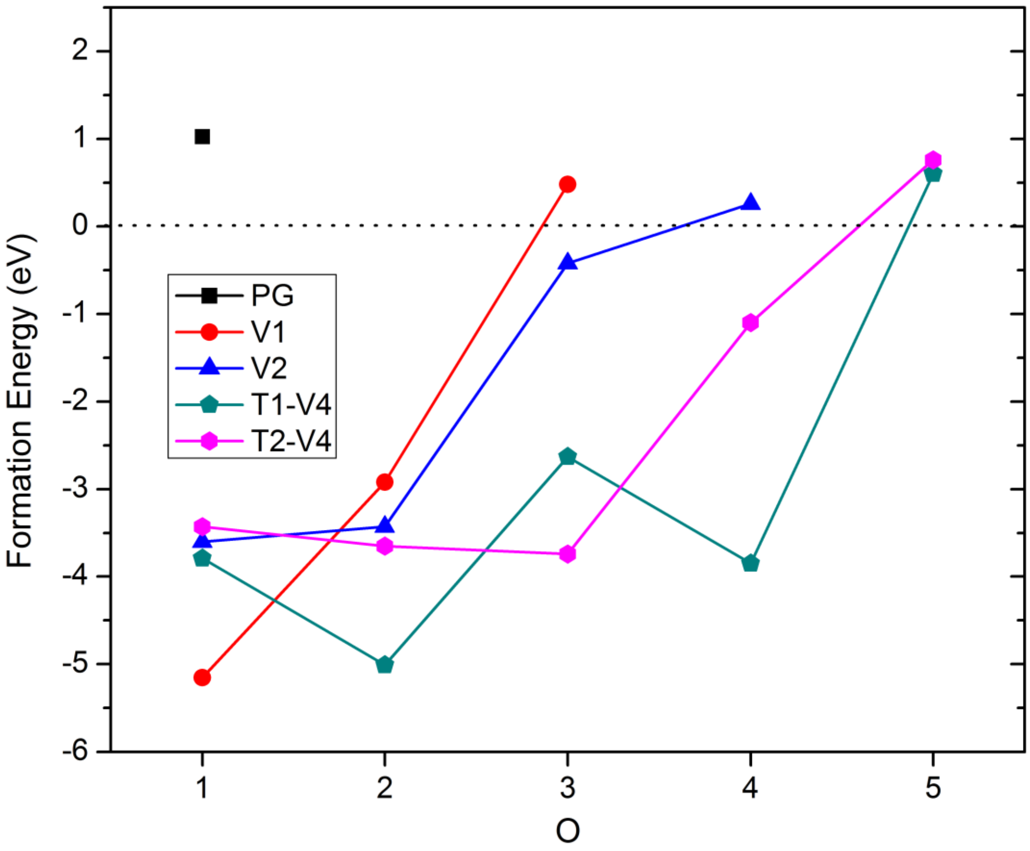

Figure S2. The oxidization energy of *ith* O of different graphene structures. The horizontal axis is the number of O atoms added.

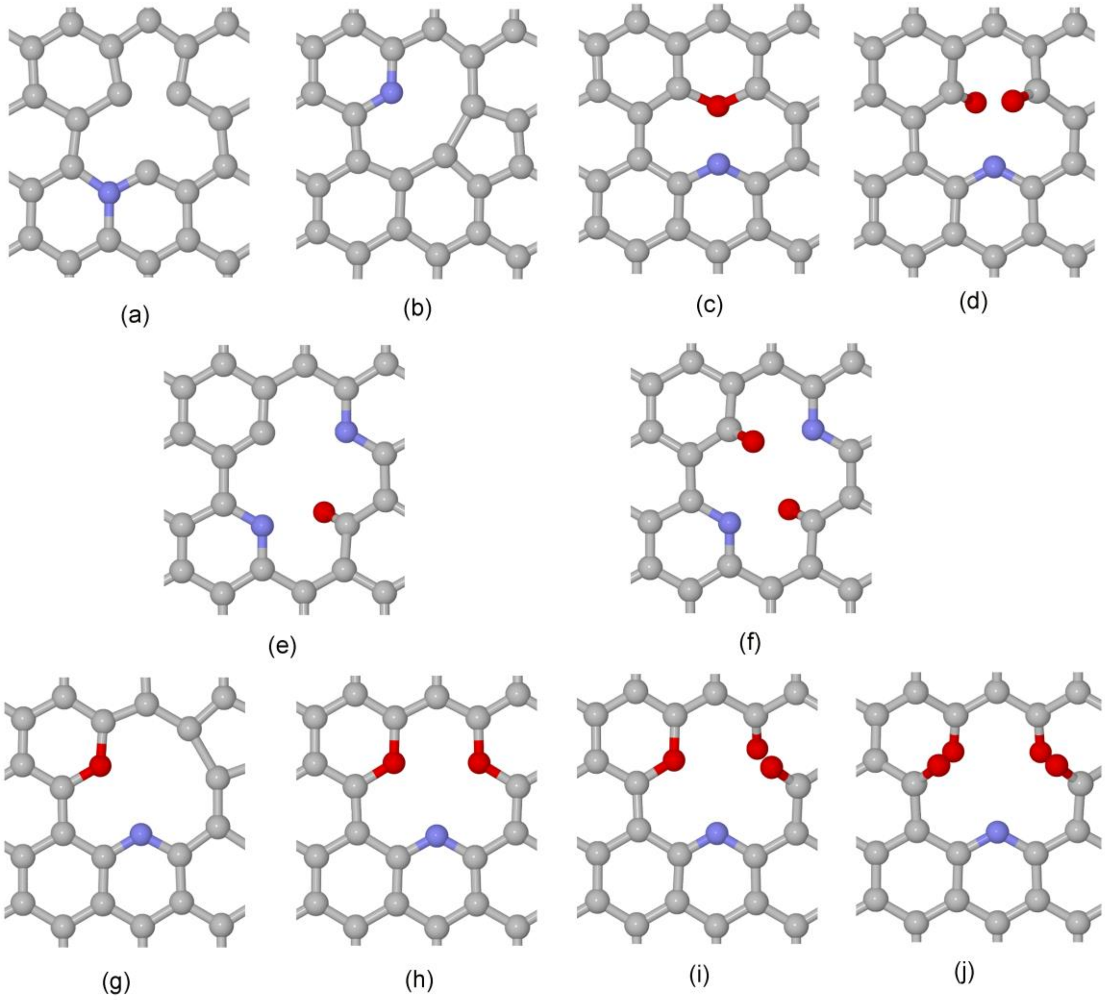

Figure S3. (a) V2-gN, (b) V2-pN, (c) V2-N1O1, (d) V2-N1O2, (e) T1-V4-N2O1, (f) T1-V4-N2O2, (g) T2-V4-N1O1, (h) T2-V4-N1O2, (i) T2-V4-N1O3, (j) T2-V4-N1O4.

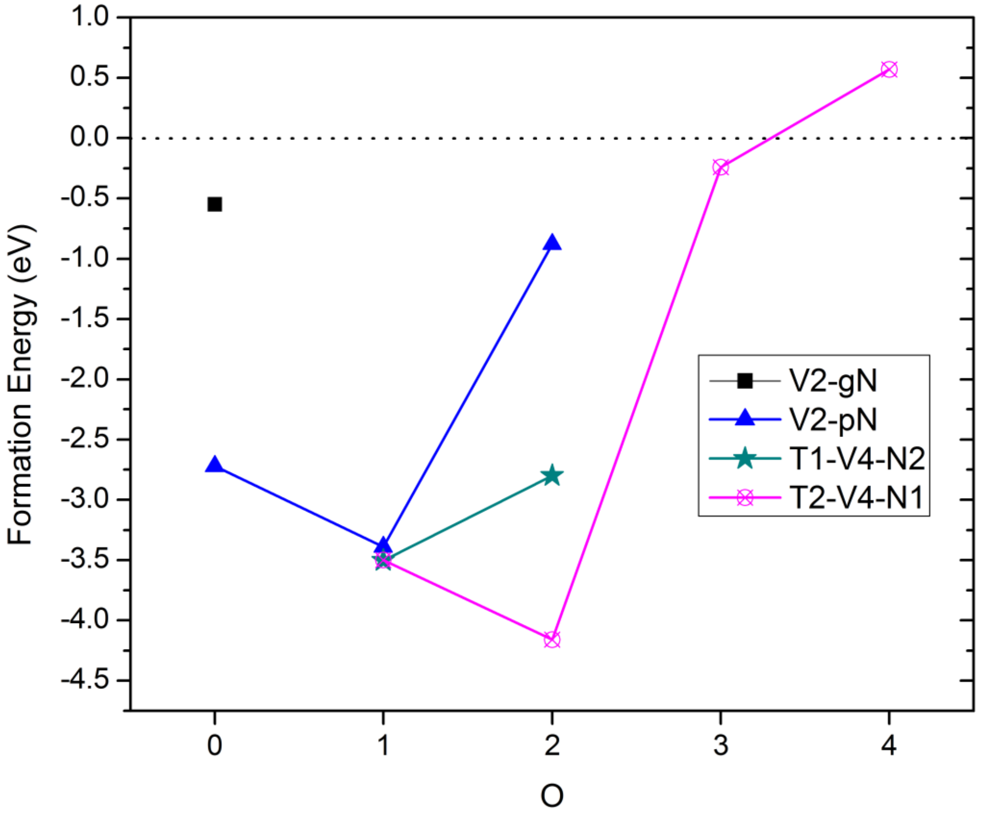

Figure S4. The N doping energy and oxidization energy of *ith* O of different graphene structures. The horizontal axis is the number of added O atoms.

SI2. Proton Transfer Profiles for Perfect Graphene

The proton transfer free energy profiles for a perfect single layer graphene were also checked and shown in **Figure S5**. The proton is assumed to approach two possible pathways, via the center of the hexatomic ring (PG-h) or via attached to a carbon atom site (PG-C) of perfect graphene. The free energy barriers are large for both PG-h (>4.0 eV) and PG-C (>3.0 eV) cases under water solution. This also indicates that the PG-C pathway is more favorable. The proton transfer barrier for PG-h was also checked by the climbing image nudged elastic band (CI-NEB) calculation using a hydronium ion for a proton, and the results are shown in **Figure S6**. The barrier calculated by NEB is about 3.0 eV. For perfect graphene, the result of NEB roughly agrees with AIMD result. For the hydrophobic surface of perfect graphene, the O-H^+ bond in hydronium need to first be broken to produce a H^+ atom and then attach to the surface site for both NEB and AIMD simulations. However, for most of the GO and N doped GO the local surface would become more hydrophilic, which would allow proton relay between water molecules from the solution to the local surface site. For AIMD simulations, the relaying path makes the proton transfer barrier significantly decreased compared with perfect graphene. For NEB simulations without water solution, the proton transfer via GO and N doped GO still like the case of perfect graphene that with O-H^+ bond breaking, and it leads to an attachment/detachment barrier of several eV for almost all the structures. Hence, we will not further discuss the problematic pathway for NEB calculations without water solution for proton transfer via GO and N doped GO.

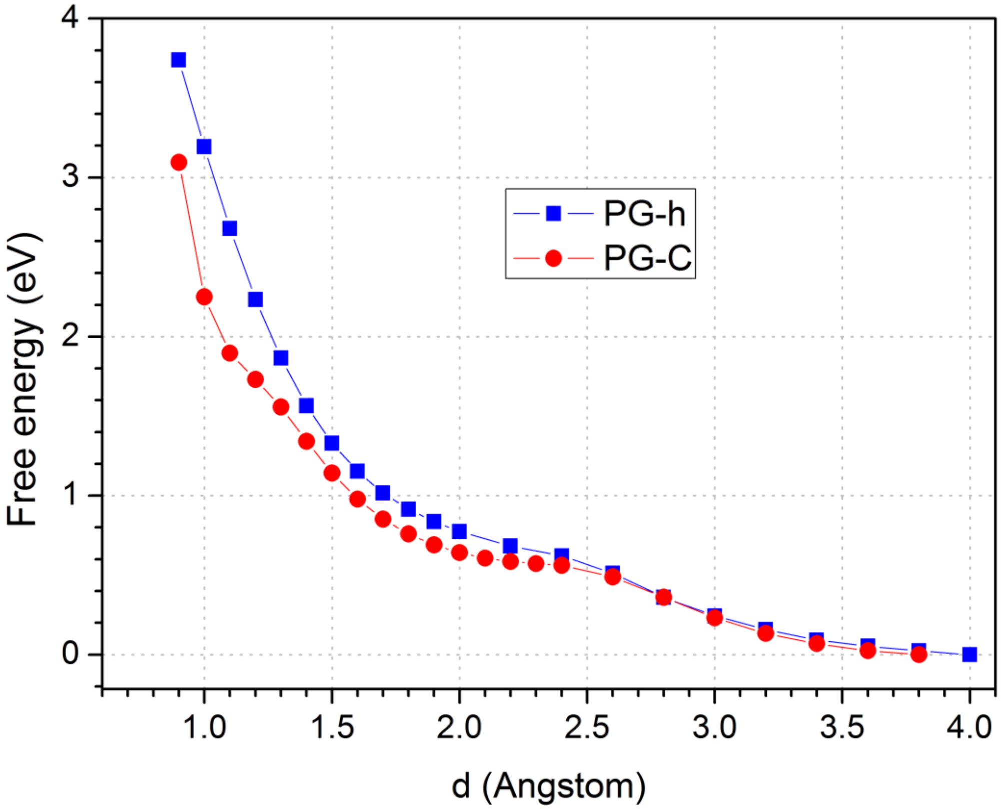

Figure S5. Proton transport free energy profiles for PG-h and PG-C structures.

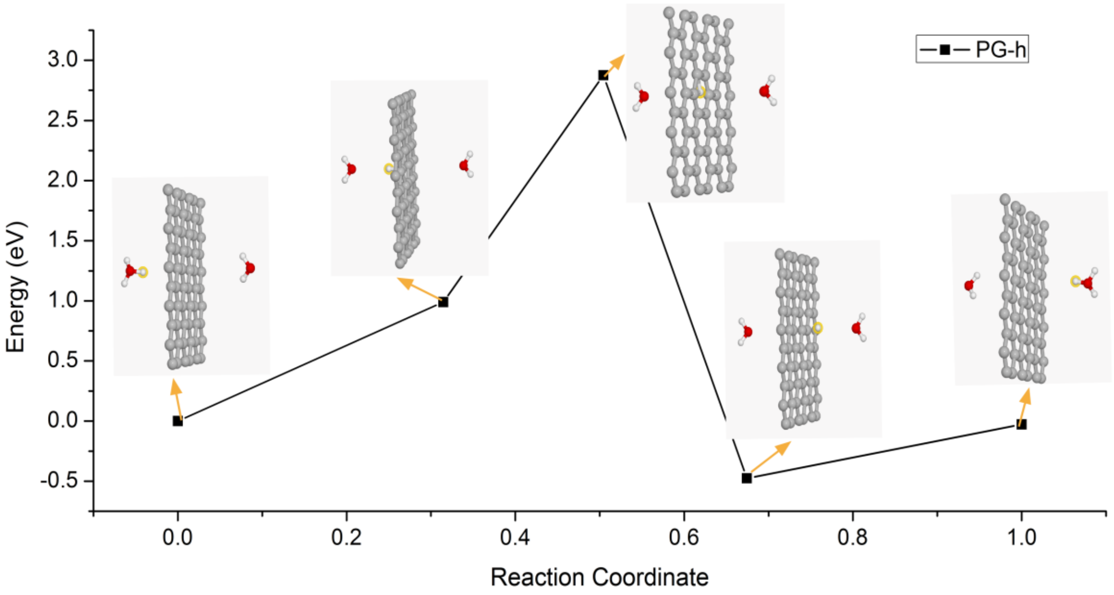

Figure S6. Proton transfer barrier for PG-h structure by climbing image nudged elastic band (CI-NEB) calculation.

SI3. Proton Transfer Profiles for V1-O2, V2-O3 and T2-V4-O4

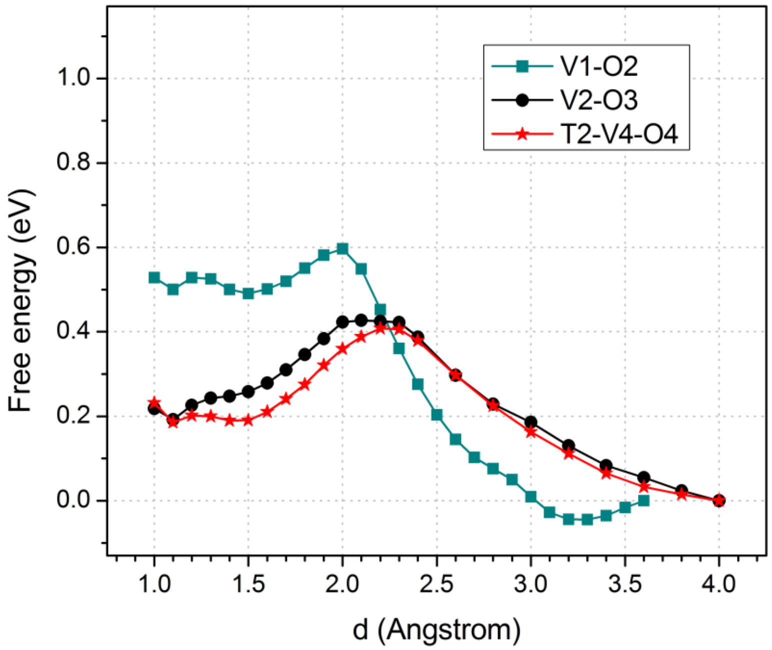

Figure S7. Proton transfer profiles for V1-O2, V2-O3 and T2-V4-O4 structures.

SI4. Pathways for Proton Transport via V2-N1O2 Structure

For the V2-N1O2 structure, the two ketone-like oxygens toward two opposite sides of the graphene plane and the doped nitrogen is in the plane as shown **Figure 2(f)** of the main text. However, the proton does not directly transfer to the N site as expected. Here, the details of the most favorable pathway for proton transfer via V2-N1O2 structure is shown in **Figure S8**. As shown in this figure, the proton is a hydronium ion when it is far away from the membrane surface. As the hydronium ion approaching the surface, the proton first relayed from the hydronium ion to the ketone-like oxygen in one side of the surface. Then, this proton continues

to relay from the ketone-like oxygen site to N site which is in the surface plane. Due to the symmetry of this structure, the proton transfer to the opposite side of the membrane is the reverse process, i.e. the proton on the N site would first relay to the ketone-like oxygen and then transfer to the solution in the opposite site. The attachment and detachment barriers for MV-1N2O(k) are 0.31 eV and 0.32 eV, respectively. This is also shown in main text.

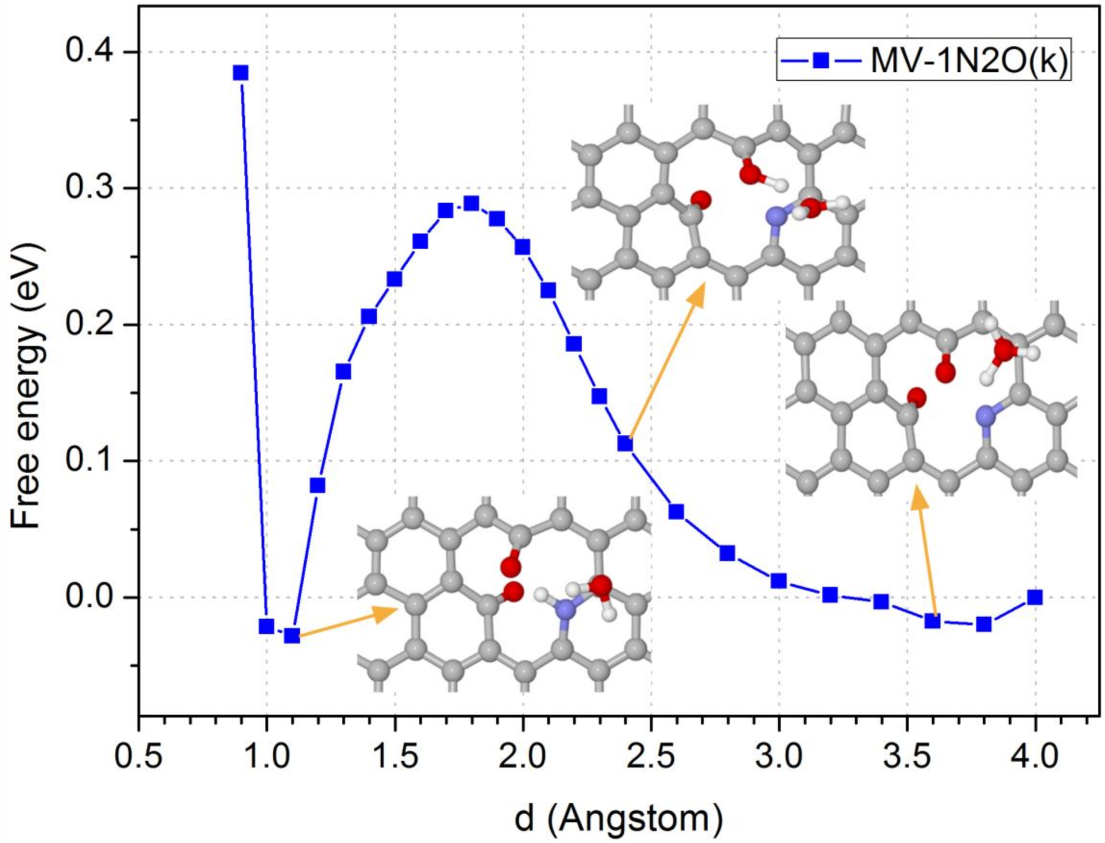

Figure S8. Proton transport free energy profile for proton approaching the N site of the V2-N1O2 structure. The geometry structures of some important points along the transport pathway are also presented. Other water molecules are removed to make the structures clear. Actually the proton relays between water molecules from water solution to the surface of the membrane.

SI5. H_2/O_2 Molecules Crossover Barriers for T1-V4-N2O2H1 Structure

The barriers for molecule crossover are checked for the T1-V4-N2O2H1 structure. The H_2/O_2 crossover barriers were calculated and presented in **Figure S9**. It can be seen that the barriers are higher than 2.36 eV and 8.0 eV for H_2 and O_2, respectively. The O_2 molecule would decompose after the distance is smaller than 1.0 Angstrom. Therefore, the molecules are almost impossible to crossover the membranes with such a small vacancy.

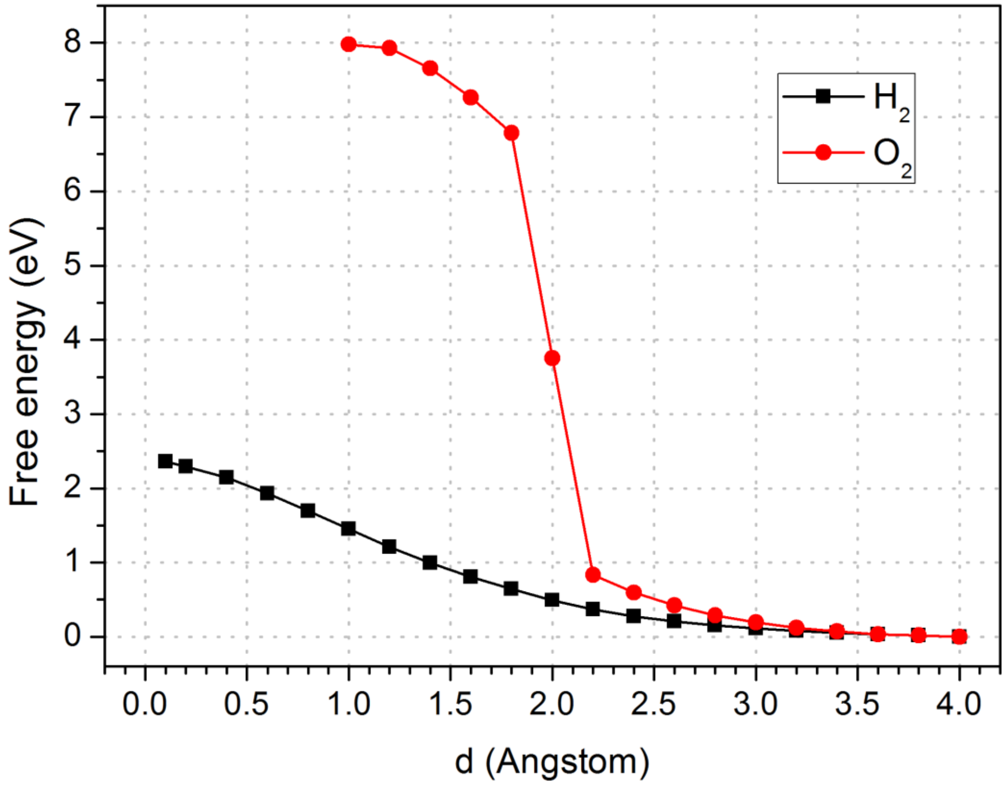

Figure S9. H_2/O_2 crossover free energy profile for the T1-V4-N2O2H1 structure. The distance between the center of molecule and center of the two N atoms in the surface is constraint.

SI6. Pathways for Proton Transport via T2-V4-N1O2 Structure

For symmetric structures investigated, the proton transfer barriers are for proton transfer in solution of one side attaching to the target sites in the membrane surface. The corresponding barriers from opposite is not calculated because it should be the same due to the symmetry. If we extrapolate the complete transfer process, the barriers should be symmetrical for an attached proton to detach from the other side of the membrane. The figures for the complete permeation process for T2-V4-N1O2 structure are shown in **Figure S10**, from which we can see that the proton is easy to be trapped to the N site due to the large detachment barrier.

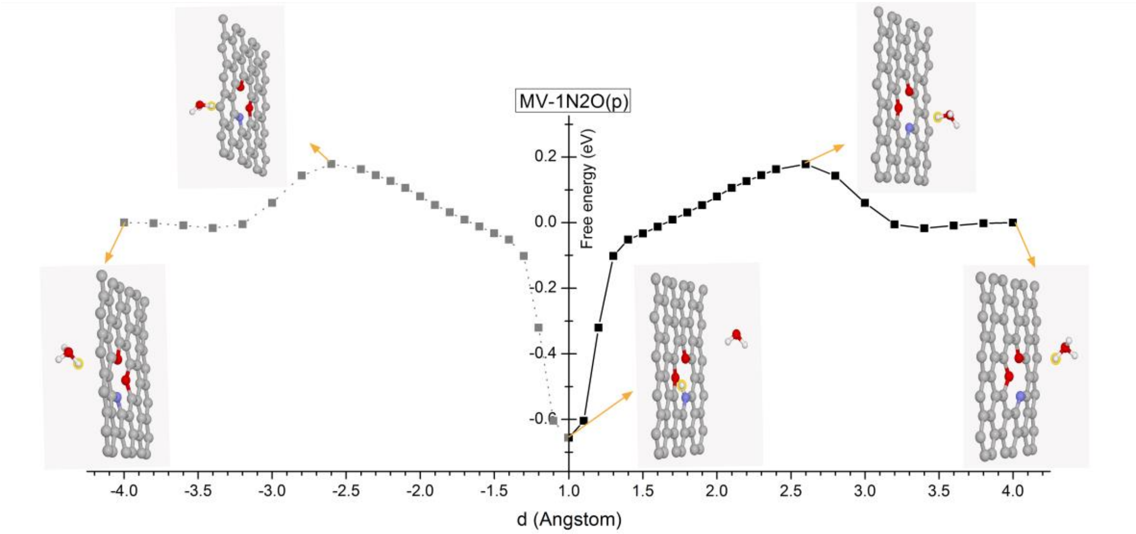

Figure S10. Proton transport free energy profile and figures for the complete permeation process for T2-V4-N1O2 structure. The solid line is for calculated barrier, and the dash line is

extrapolated to the other side due to the symmetry. The “d” is distance between proton and N atom.

SI7. Discussions for formation of V2-N1O2 structure

As indicated by previous study, the pyridinic N doped mono-vacancy is the most popular structures observed in experiments.[1] In the present study, this structure corresponding to V2-pN. Under an environment with O_2, the V2-pN structure can be oxidized to a *cis*-V2-N1O2 structure with a low barrier of 0.40 eV as shown in **Figure S11**. The two O atoms of the formed cis-V2-N1O2 structure are in the same side of graphene, which is thermodynamically less stable than the structure with two O atoms in opposite side *trans*-V2-N1O2 structure. The *cis*-V2-N1O2 structure will transfer to the *trans*-V2-N1O2 structure with a barrier about 0.68 eV as shown in **Figure S12**. And the total energy of *trans*-V2-N1O2 structure is 0.60 eV lower than *cis*-V2-N1O2 structure. However, further oxidization of *trans*-V2-N1O2 structure is rather difficult as shown in **Figure S11**. If another O_2 approaching the highlighted C site of the V2-N1O2 structure, one CO_2 is formed with a high barrier of about 4.0 eV. Hence, the V2-N1O2 structure is rather stable.

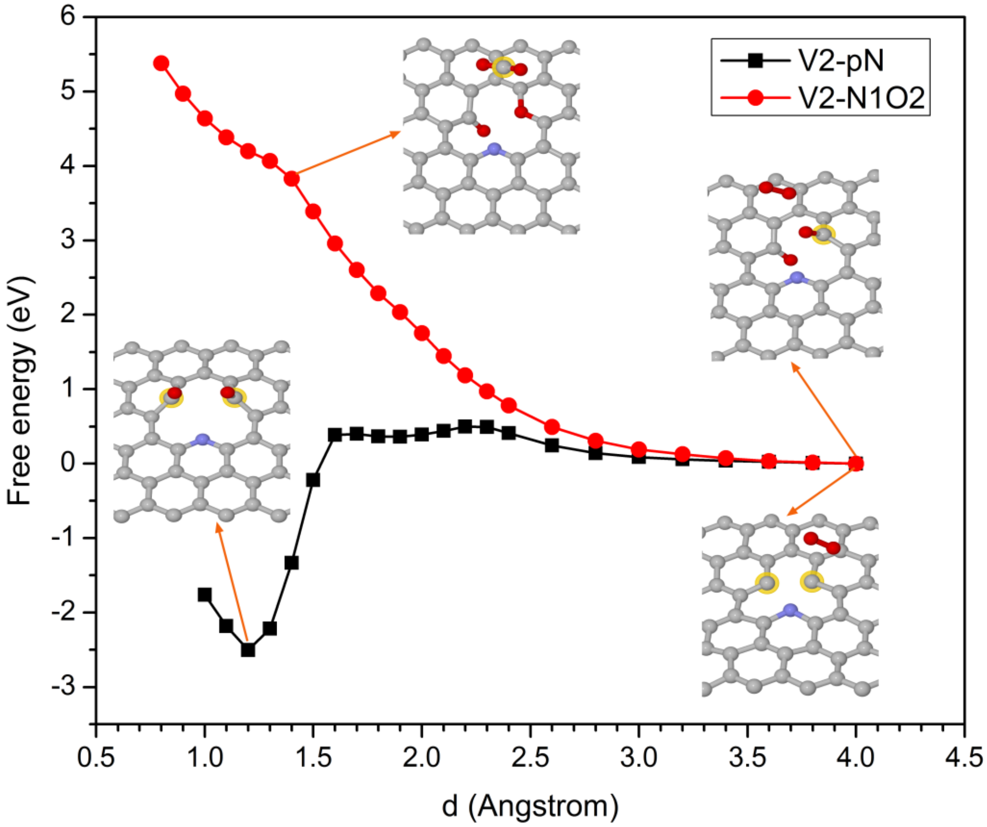

Figure S11. The oxidization energies barriers for V2-pN and V2-N1O2 structures. The distance along horizontal axis is the distance between the center of an O_2 molecule and the highlighted C atom (for V2-N1O2 structure) / center of the highlighted two C atoms (for V2-pN structure).

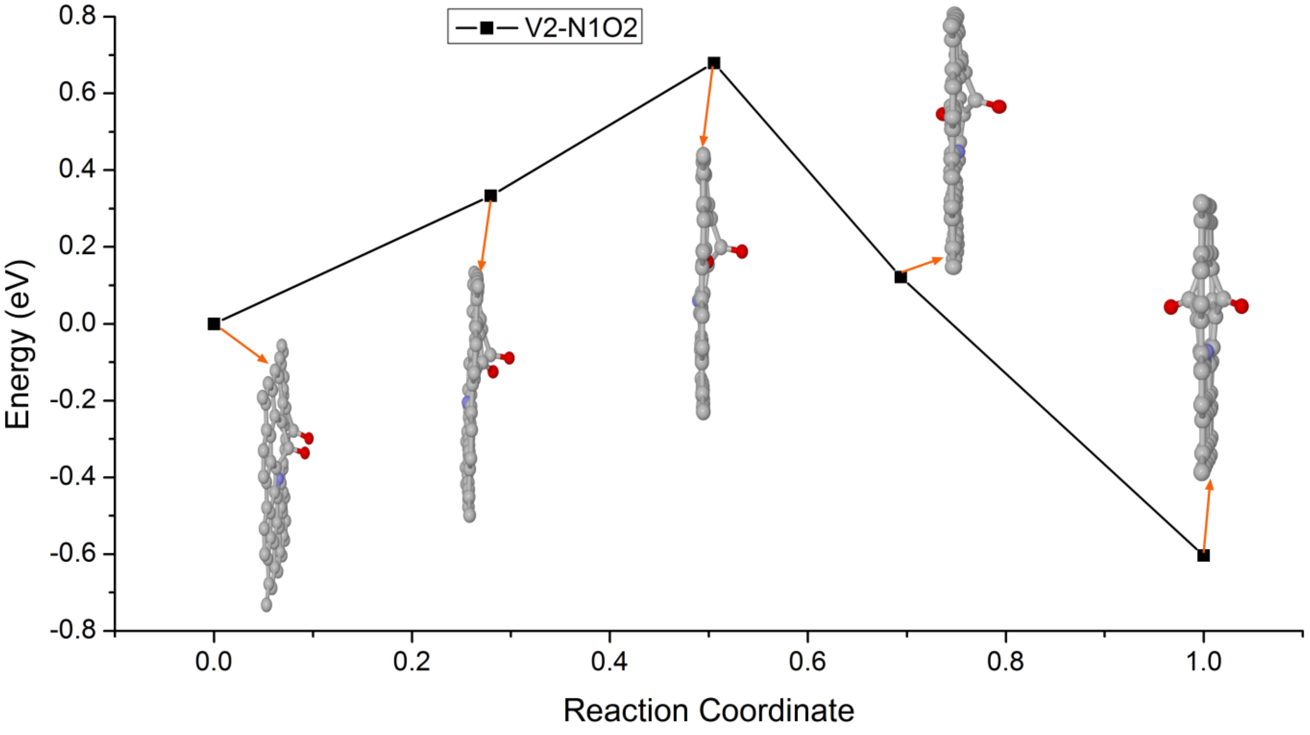

Figure S12. Structure transfer barrier from *cis*-V2-N1O2 structure to *trans*-V2-N1O2 structure by climbing image nudged elastic band (CI-NEB) calculation.

SI8. Convergence of calculated force

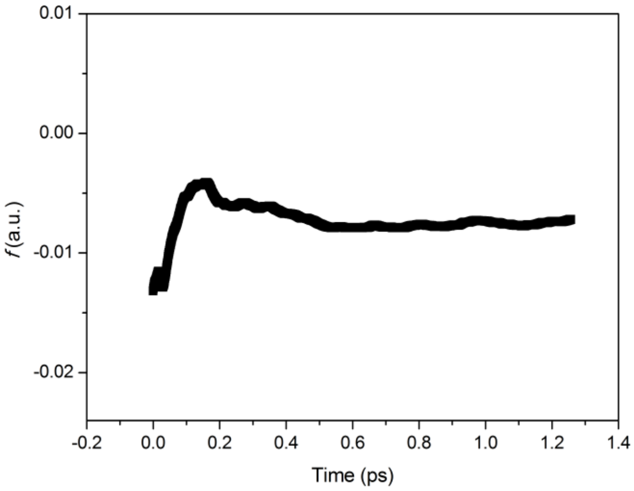

Figure S13. The average force VS. time steps for V2-N1O2 structure with constraint distance of 1.3 Angstrom.

References

1. Kondo, T. *et al.* Atomic-scale characterization of nitrogen-doped graphite: Effects of dopant nitrogen on the local electronic structure of the surrounding carbon atoms. *Phys. Rev. B* **86**, 035436 (2012).
